# miR-144-3p inhibits cell proliferation of colorectal cancer cells by targeting BCL6 via inhibition of Wnt/β-catenin signaling

**DOI:** 10.1186/s11658-020-00210-3

**Published:** 2020-03-17

**Authors:** Naihui Sun, Liang Zhang, Chongguang Zhang, Yuan Yuan

**Affiliations:** 1 Department of Surgery, Ninghe Hospital, Tianjin, 301500 China; 2 Department of Retired Office, North China University of Science and Technology, No. 46, Xinhuaxi road, Tangshan, 063000 Hebei China

**Keywords:** miRNA, miR-144-3p, BCL6, Wnt/β-catenin, CRC

## Abstract

**Background:**

It has been shown that miR-144-3p regulates cell proliferation, apoptosis, migration and invasion in various cancers. However, the function and expression of miR-144-3p in colorectal cancer (CRC) remained obscure.

**Methods:**

Immunohistochemical (IHC) staining was performed to investigate the protein expression of BCL6 in CRC tissues. The effect of BCL6 and miR-144-3p on CRC cells was explored through methylthiazolyl tetrazolium (MTT) assay, colony formation and cell cycle assays. Luciferase reporter assays, reverse transcription quantitative polymerase chain reaction (RT-qPCR) and Western blot assay were carried out to determine that BCL6 is directly regulated by miR-144-3p.

**Results:**

Our results showed that miR-144-3p is down-regulated in CRC and correlates with the tumor progression of CRC patients. miR-144-3p inhibits cell proliferation and delays G1/S phase transition of CRC cells. Moreover, we found that BCL6 is a new target of miR-144-3p. Furthermore, BCL6 is a mediator of miR-144-3p repression of cell proliferation and cell cycle arrest in CRC cells. miR-144-3p repression of Wnt/β-catenin signaling is mediated by BCL6 in CRC cells.

**Conclusions:**

Overall, the effect of the miR-144-3p/BCL6 axis on regulating CRC carcinogenesis was demonstrated, and miR-144-3p was identified as a potential prognostic and therapeutic target in colorectal cancer.

## Background

MicroRNAs (miRNAs) are a class of noncoding RNAs ranging from 18 to 24 nt in length with involvement in endogenous regulation of post-transcriptional gene expression, resulting in gene silencing generally [1]. More and more evidence has indicated that miRNAs exhibit dysregulation in the diverse developmental process of different types of human cancer, suggesting that miRNAs play a vital role in different biological processes including cell proliferation, development, metastasis, apoptosis, and cell cycle progress [2–5].

Recent reports show that abnormal expression of miR-144-3p plays either tumor suppressor or oncogene roles in various cancers. For instance, miR-144-3p targets and regulates the expression of connexin 43, leading to the suppression of bone formation in distraction osteogenesis [6]. miR-144-3p represses epithelial-to-mesenchymal transition of gastric cancer by down-regulating PBX3 [7]. miR-144-3p exerts the effect of a tumor suppressor on glioblastoma through regulating c-Met [8]. Moreover, in hepatocellular carcinoma (HCC), miR-144-3p acts as a tumor suppressor microRNA to regulate the progression of HCC [9, 10]. In pancreatic cancer, MiR-144-3p inhibits cell proliferation, migration, and invasion through targeting AP-1 transcription factor subunit (FOSB) and promotes cell apoptosis and cell cycle arrest by targeting proline-rich protein 11 [11, 12]. Furthermore, miR-144-3p presents an oncogene role in clear cell renal cell carcinoma (ccRCC) by regulating ARID1A [13]. However, the function of miR-144-3p in CRC remained unknown.

The BCL6 gene encodes a 95-kD nuclear phosphor protein belonging to the BTB/POZ/zinc finger (ZF) family of transcription factors [14]. BCL6 has been shown to modulate the expression of genes involved in B cell activation, differentiation, cell cycle arrest, and apoptosis [15–18]. In addition, BCL6 could target the transcription of two critical oncogenes, MYC and BCL2, leading to MYC deregulated expression to regulate cell growth [19]. A previous study showed that BCL6 targets many genes belonging to multiple functional pathways including toll-like receptors, INF-R, a variety of cytokines, TGF-R, and WNT signaling [20]. We found that miR-144-3p inhibited cell proliferation of colorectal cancer cells. Moreover, β-catenin as a therapeutic target for colon cancer led us to explore the role of miR-144-3p in the Wnt/β-catenin signaling pathway [21]. However, the role of BCL6 in CRC remained unclear.

Herein, we found that that miR-144-3p is down-regulated in CRC and correlated with the tumor progression of CRC patients. We demonstrated that miR-144-3p inhibits cell proliferation and delays G1/S phase transition of CRC cells. Moreover, we found that BCL6 is a new target of miR-144-3p. Furthermore, BCL6 is a mediator of miR-144-3p repression of cell proliferation and cell cycle arrest in CRC cells. miR-144-3p repression of Wnt/β-catenin signaling is mediated by BCL6 in CRC cells. Together, our findings may provide a new molecular biomarker and therapeutic target to shed light on the carcinogenic mechanism of colorectal cancer.

## Methods

### Clinical CRC specimens

Twenty pairs of CRC tissues and adjacent non-tumor tissues were collected from Tianjin Ninghe Hospital. This study was performed in accordance with the ethical standards of the institutional committee.

### Cell culture and transfection

HCT116 and SW480 cells were cultured in RPMI 1640 medium supplemented with 10% fetal bovine serum (FBS), 100 mg/mL streptomycin, and 100 IU/mL penicillin. HCT116 cells were maintained in a humidified incubator with 5% CO_2_ at 37 °C. All the transfection was performed with Lipofectamine 2000 transfection reagent (Invitrogen, Carlsbad, CA, USA) according to the manufacturer’s protocol.

### RNA extract, quantitative reverse transcript-PCR (qRT-PCR), and western blot analysis

Total RNA was isolated from CRC tissues and cells with the mirVana miRNA Isolation Kit (Ambion, Austin, TX, USA) according to the manufacturer’s instructions. The RNAs were reversely transcribed into cDNAs with random primers and the miRNA inverse transcription primers: miR-144-3p RT: 5′-GTCGTATCCAGTGCAGGGTCCGAGGTGCACTGGATACGACAGTACA-3′; U6-RT: 5′-GTCGTATCCAGTGCAGGGTCCGAGGTGCACTGGATACGACAAAATATGG-3′. The following primers were designed for PCR detection:

- miR-144-3p-S, 5′-TGCGGTACAGTATAGATGAT-3′,
- miR-144-3p-AS, 5′-CCAGTGCAGGGTCCGAGGT-3′;
- U6-S, 5′-TGCGGGTGCTCGCTTCGGCAGC-3′, U6-AS,5′-CCAGTGCAGGGTCCGAGGT-3′;
- BCL6-S, 5′-TCCTCGGAAGATGAGATTGC-3′,
- BCL6-AS, 5′-GTTGAGCACGATGAACTTGTA-3′;
- β-actin-S, 5′-CTACGTCGCCCTGGACTTCGAGC-3′,
- β-actin-AS, 5′-GATGGAGCCGCCGATCCACACGG-3′. The detailed experimental protocol of RNA extraction and RT-qPCR can be found in ref. [22].

### Vector construction

MiR-144-3p mimics, the miR control, the overexpression plasmid of BCL6, 3′UTR and 3′UTR mutant of BCL6 gene expression plasmids were purchased from Invitrogen (Carlsbad, CA).

### Luciferase reporter assay

HCT116 cells were seeded on a 48-well plate and co-transfected with either miR-144-3p mimics or miR control together with the reporter plasmid including either wild-type or mutant 3′UTR of BCL6. The fluorescence detection used a Luciferase Assay System (Promega, USA) 48 h after transfection according to the manufacturer protocols.

### MTT and colony formation assays

HCT116 and SW480 cells were transfected with miR-144-3p mimics or miR control, BCL6 overexpression or inhibition vector, with transfection after 24 h to perform MTT and colony formation assays. Details of the procedure of MTT and colony formation assays were described by Zhao et al. [23].

### Cell cycle analysis by flow cytometry

HCT116 cells were transfected with miR-144-3p mimics or miR control, co-transfected with either miR-144-3p mimics or miR control together with BCL6 overexpression vector, and 24 h after transfection, cell cycle distribution of HCT116 cells were examined by flow cytometry analyses, which were performed using annexin V and propidium iodide staining according to [23, 24].

### Immunohistochemistry

Twenty pairs of CRC tissues and adjacent non-tumor tissues were fixed in 4% formaldehyde for 24 h and sent to Tianjin Saier Co. for immunohistochemistry.

### Statistical analysis

All statistical analyses were performed at least three times for independent experiments. Data are presented as mean ± SD and were analyzed with two-tailed unpaired Student’s t-test or ANOVA analysis to make the comparison. *P* ≤ 0.05 was considered statistically significant.

## Results

### Expression of miR-144-3p is down-regulated in CRC tissues and related to the clinicopathologic characteristics of patients

**Fig. 1:**
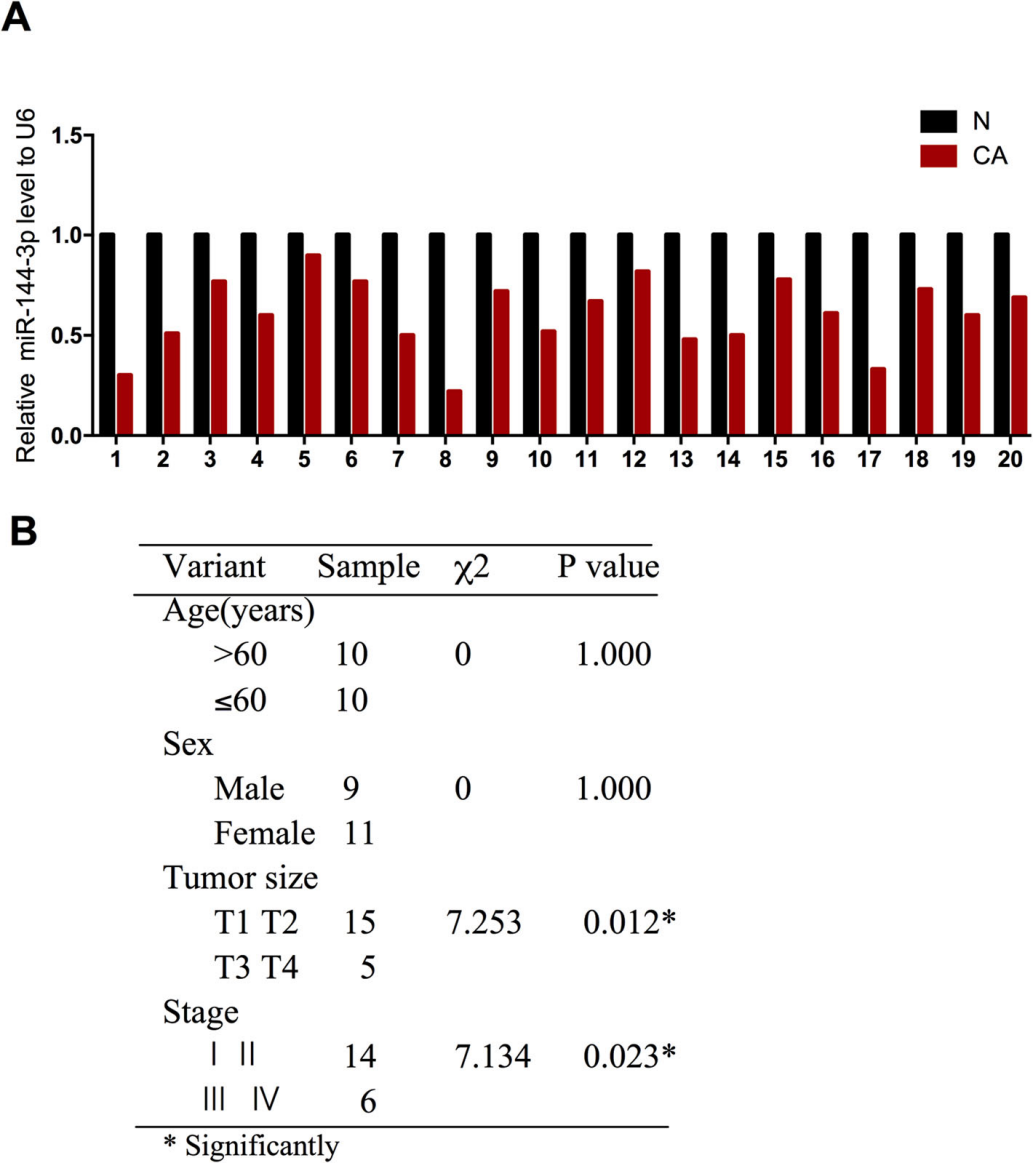
Expression of miR-144-3p level in CRC tissues and related to the clinicopathologic characteristics of patients. **a** qRT-PCR showed the expression levels of miR-144-3p in 20 pairs of CRC tissues and adjacent non-tumor tissues, U6 snRNA was used as an internal control. **b** Correlation between miR-144-3p and clinicopathologic characteristics of patients with CRC

To explore the potential role of miR-144-3p in the progression of CRC, we examined the level of miR-144-3p in 20 CRC tissues and non-tumor adjacent tissues using qRT-PCR. Figure 1a shows that miR-144-3p was significantly down-regulated in CRC tissues when compared to non-tumor adjacent tissues. Moreover, we analyzed miR-144-3p expression with clinicopathologic characteristics of patients in CRC tissues. As shown in Fig. 1b, higher miR-144-3p expression was associated with a lower stage of CRC. These results indicate that miR-144-3p is down-regulated in CRC and correlates with the tumor progression of CRC patients.

### miR-144-3p inhibits cell proliferation and delays G1/S phase transition in CRC cells

**Fig. 2:**
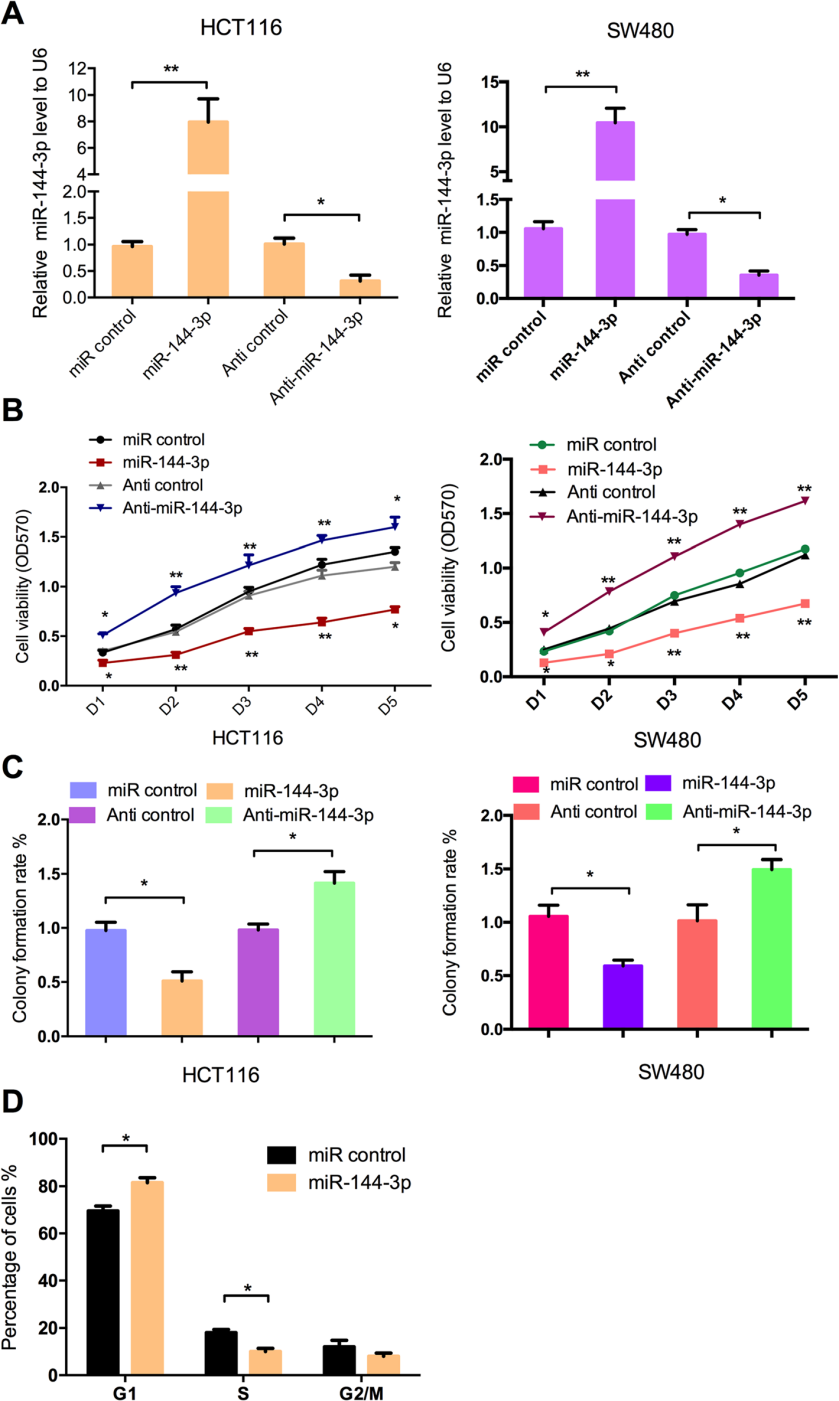
miR-144-3p inhibits cell proliferation and delays G1/S phase transition in CRC cells. (**a**) qRT-PCR showed the expression of miR-144-3p mimics in HCT116 and SW480 cells. MTT assay (**b**) and colony formation assay (**c**) were performed to detect the effect of miR-144-3p on cell proliferation activity in CRC cells. (**d**) Flow cytometry analysis was performed to determine the distribution of cells in the cell cycle of HCT116 cells

To determine whether miR-144-3p plays a role in CRC cells, HCT116 and SW480 cells were transfected with miR-144-3p mimics, anti-miR-144-3p or the negative control accordingly. In further analyses, the efficiency of these vectors was validated by qRT-PCR (Fig. 2a). Then, MTT and colony formation assay were performed. The results showed that overexpression of miR-144-3p inhibited cell proliferation of CRC cells, while anti-miR-144-3p promoted CRC cell proliferation (Fig. 2b, c). In order to study the underlying mechanism, flow cytometry analysis was performed to detect the cell cycle progression. The results showed that miR-144-3p inhibited the G1/S transition of HCT116 cells (Fig. 2d). Taken together, the results indicate that miR-144-3p inhibits cell proliferation and delays G1/S phase transition in CRC cells.

### BCL6 is a target of miR-144-3p

**Fig. 3:**
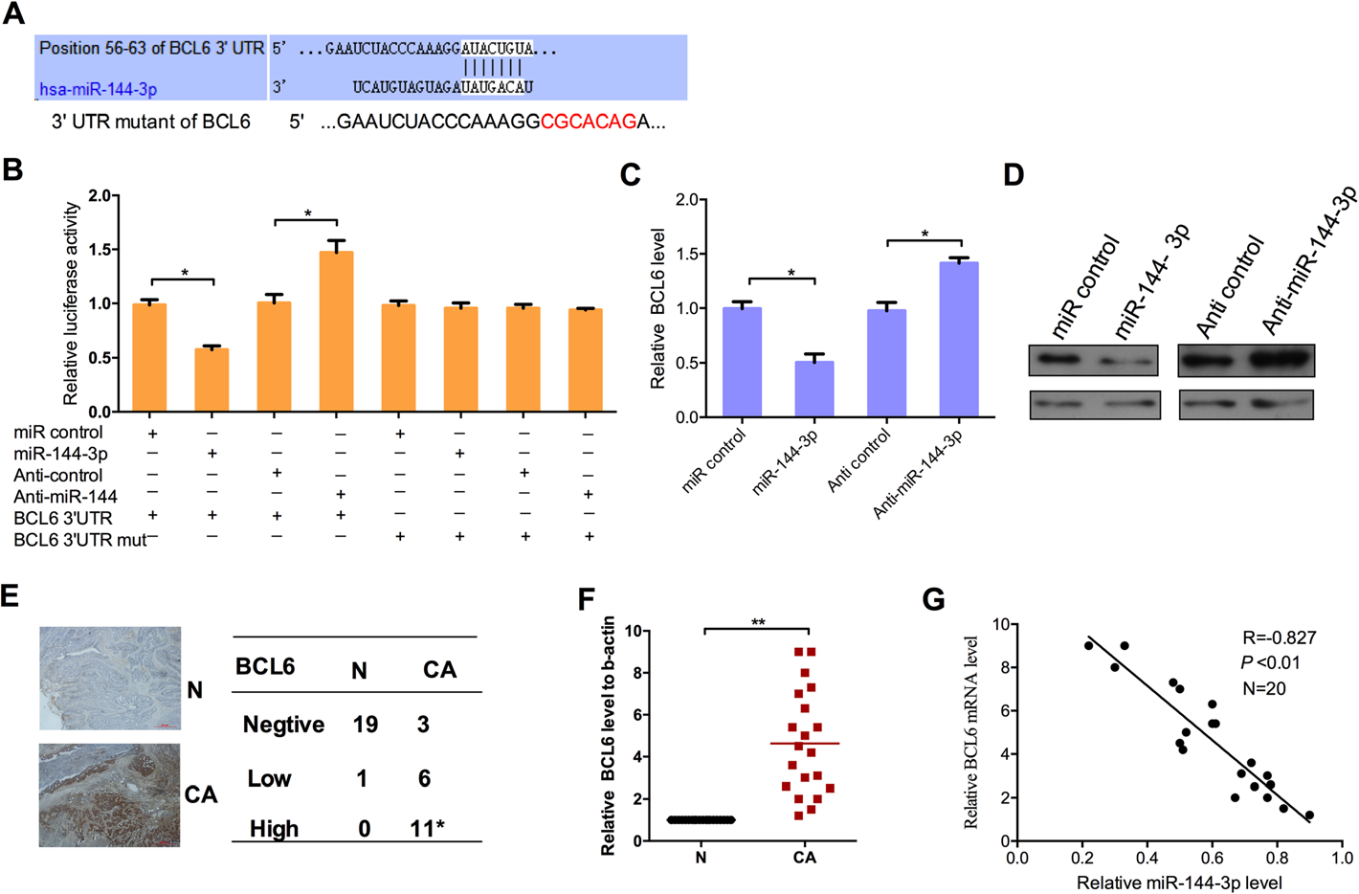
BCL6 is a target of miR-144-3p. (**a**) The predicted binding sites for miR-144-3p in the 3′UTR of BCL6 and the mutant 3′UTR of BCL6 are shown. (**b**) Luciferase reporter assay was performed in HCT116 cells co-transfected miR-144-3p mimics or the control vector with BCL6–3′UTR1, BCL6–3′UTR1-mut. qRT-PCR (**c**) and Western blot (**d**) were performed to detect the mRNA and protein levels of BCL6 in HCT116 cells transfected with miR-144-3p mimics or the control vector. (**e**) Immunohistochemical staining with BCL6 in CRC tissue samples and the representative graph of expression levels of BCL6. Original magnification: 400Χ. (**f**) qRT-PCR showed the expression levels of BCL6 in 20 pairs of CRC tissues and adjacent non-tumor tissues. β-actin was used as an internal control. (**g**) Pearson’s correlation analysis indicated a negative correlation between the expression level of miR-144-3p and BCL6 in 20 pairs of CRC tissues (R = − 0.827; **: *P* < 0.01)

Using bioinformatics analyses TargetScan 7.2, RNAhybrid (v2.2), PITA Catalog version 6 and miRanda (v1.9) to predict the target of miR-144-3p, among them, BCL6 was chosen for further study due to its high scores. We identified putative binding sites for miR-144-3p within the 3′UTR of BCL6 mRNA. In order to validate whether BCL6 is targeted by miR-144-3p, a human wild type or the mutant sites 3′UTR of the BCL6 fragment was cloned into luciferase reporter plasmids (Fig. 3a). HCT116 cells were co-transfected with either miR-144-3p mimic, anti-miR-144-3p or the negative control vector with the wild type or the mutant sites 3′UTR of the BCL6 overexpression vector, as shown in Fig. 3b. Compared with the negative control group, fluorescence intensity of the wild type BCL6 3′UTR was significantly reduced by overexpression of miR-144-3p, while it was increased by overexpressing the anti-miR-144-3p vector in HCT116 cells. However, the fluorescence intensity of the mutant BCL6 3′UTR was not changed by overexpression or inhibition of miR-144-3p. In addition, qRT-PCR and Western blot assay showed that ectopic expression of miR-144-3p reduced the mRNA and protein level of BCL6, while the opposite effects were observed in anti-miR-144-3p-transfected cells at both mRNA and protein levels (Fig. 3c, d).

Likewise, to explore whether miR-144-3p is relevant to endogenous BCL6 expression in human CRC, immunohistochemical staining and qRT-PCR assay were performed to determine the mRNA and protein level of BCL6 in 20 pairs of CRC tissues and non-tumor adjacent tissues. The results showed that the expression of BCL6 in CRC tissues was up-regulated at both protein and mRNA levels in comparison to the non-tumor adjacent tissues (Fig. 3e, f). Moreover, correlation analysis indicated that the expression of miR-144-3p and BCL6 had a negative correlation in the 20 pairs of CRC tissues (Fig. 3g). These results suggest that miR-144-3p negatively regulates BCL6.

### The miR-144-3p/BCL6 axis regulates malignant behavior of CRC cells

**Fig. 4:**
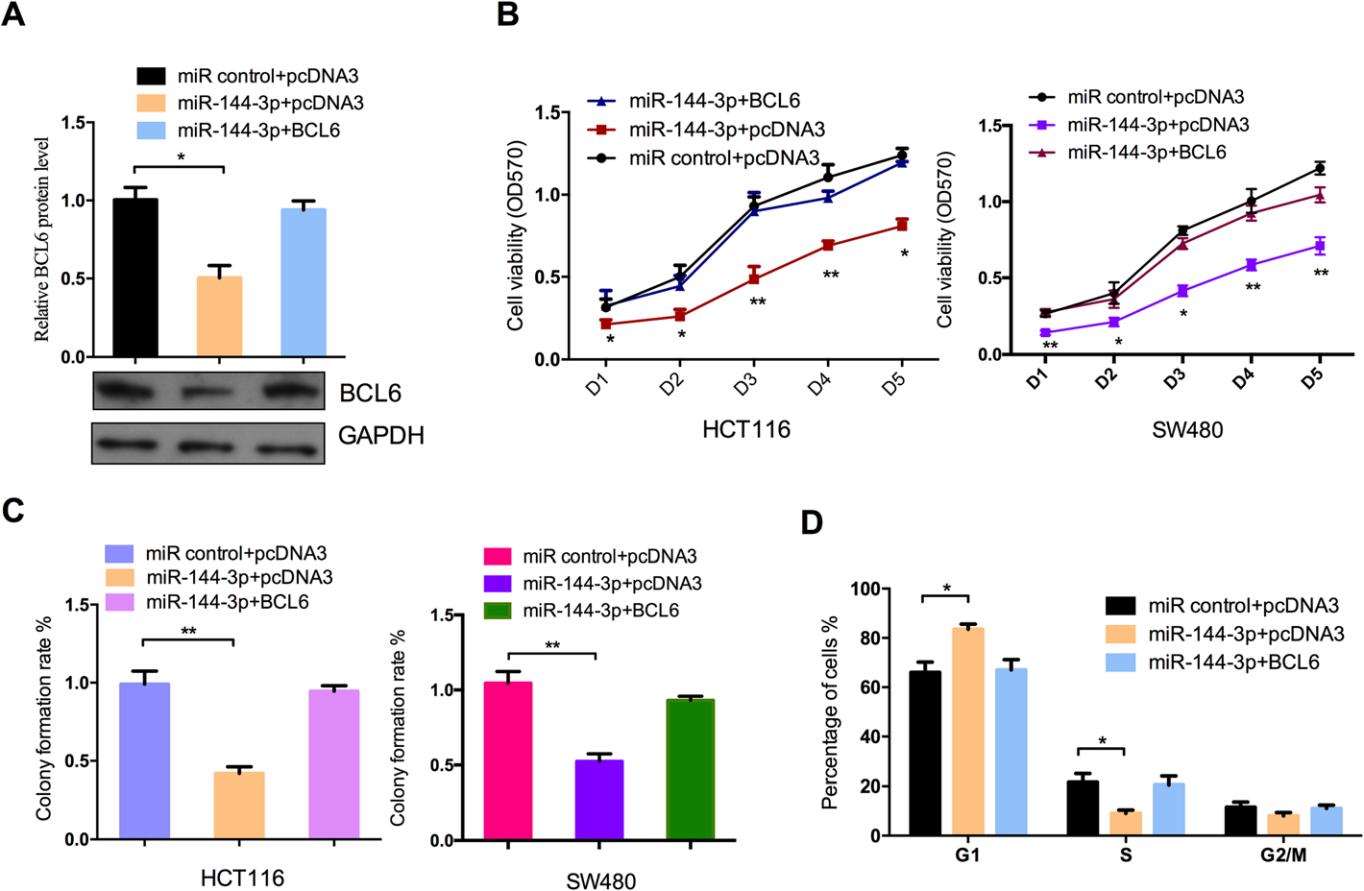
The miR-144-3p/BCL6 axis regulates malignant behavior of CRC cells. (**a**) HCT116 cells were co-transfected with miR-144-3p mimics and pcDNA3/BCL6 or the control vector. Western blot assay was carried out to detect the protein level of BCL6. CRC cells were co-transfected with miR-144-3p mimics and pcDNA3/BCL6 or the control vector. MTT assay (**b**), colony formation assay (**c**) and cell cycle assay (**d**) were performed to determine the malignant behavior of CRC cells

Furthermore, to confirm that the effect of miR-144-3p on cell proliferation and cell cycle progression of CRC cells is due to its regulation of BCL6, a series of rescue experiments were performed. Figure 4a shows that ectopic expression of miR-144-3p could reduce BCL6 protein expression in HCT116 cells, and re-expression of BCL6 overexpression plasmid was effective to restore the protein level of BCL6. In addition, the functional rescue experiments showed that miR-144-3p-mediated repression of cell proliferation and cell cycle progression in CRC cells was restored by ectopic expression of the BCL6 overexpression plasmid (Fig. 4b, c, d). These results show that BCL6 is a mediator of miR-144-3p repression of cell proliferation and cell cycle arrest in CRC cells.

### The miR-144-3p/BCL6 axis inhibits the Wnt/β-catenin signaling pathway

**Fig. 5:**
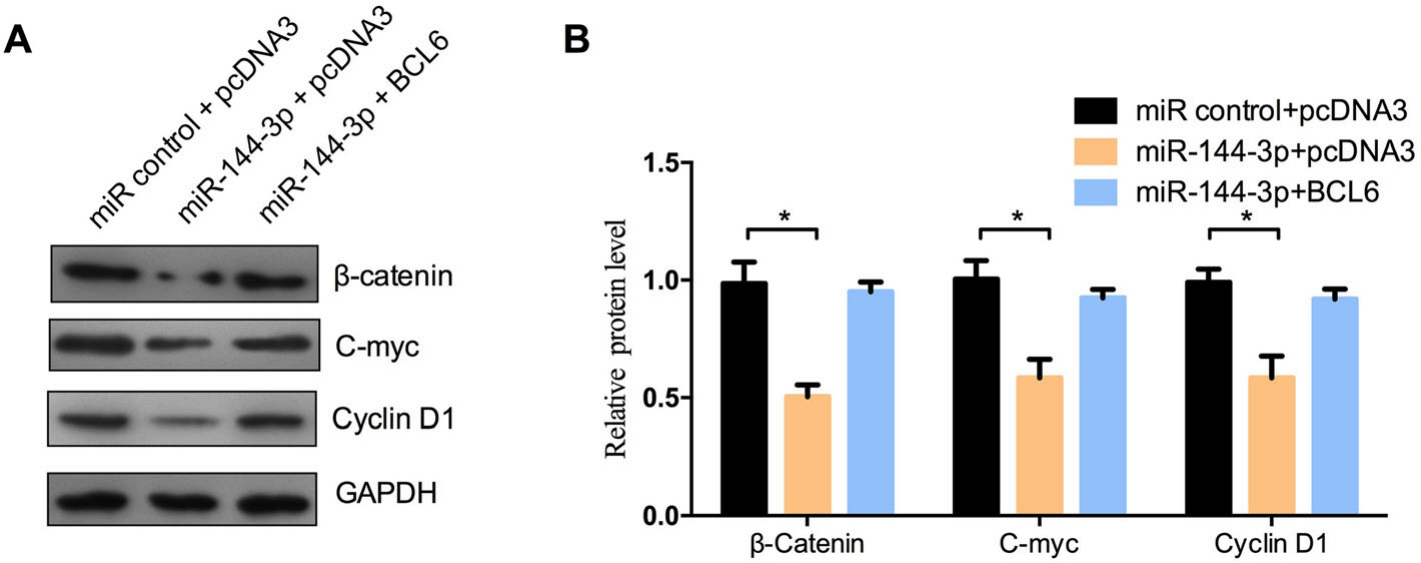
The miR-144-3p/BCL6 axis inhibited the Wnt/β-catenin signaling pathway. HCT116 cells were co-transfected with miR-144-3p mimics and pcDNA3/BCL6 or the control vector. Western blot analysis of β-catenin, C-myc and cyclin D1 protein levels in HCT116 cells

Next, we investigated the functional relevance of the interaction between miR-144-3p and BCL6 by determining the effect of changes in their expression levels on the activity of the Wnt/β-catenin signaling pathway, then the important downstream target genes (β-catenin, c-myc, cyclin D1) of the Wnt/β-catenin pathway were examined using Western blot assay (Fig. 5a, b). The results showed that miR-144-3p caused a decrease in the protein level of β-catenin, c-myc, and cyclin D1, while ectopic expression of BCL6 could restore the protein level of β-catenin, c-myc, and cyclin D1. The results indicate that the role of miR-144-3p/BCL6 repression of cell proliferation may involve Wnt/β-catenin signaling.

## Discussion

Colorectal cancer (CRC) is one of the most common cancers worldwide. With 600 000 patients dying from colorectal cancer annually and with an unfavorable prognosis of advanced CRC [25], it is urgent to explore and identify new biomarkers for diagnosis and treatment in CRC. Recent reports indicate that miRNAs play a vital role in the development and progression of various cancers [1]. Herein, our findings indicated that miR-144-3p was down-regulated in CRC and correlated with the tumor progression of CRC patients. Furthermore, we showed that miR-144-3p inhibited cell proliferation and delayed G1/S phase transition of HCT116 cells. It may manifest the tumor suppressor of miR-144-3p in CRC.

miRNA plays its role via regulating the expression of its target gene [26]. B-cell lymphoma 6 (BCL6), also known as BCL6A, LAZ3, or ZNF51, belonging to the BTB-POZ protein family, has been demonstrated to facilitate cell proliferation [17]. Reports showed that BCL6 may be a novel diagnostic and treatment strategy for breast or ovarian cancer [27, 28]. However, the role BCL6 plays in CRC remained unknown. In this study, we used bioinformatics analyses to determine that BCL6 is a potential target of miR-144-3p based on functional knowledge of miR-144-3p. Then we demonstrated that BCL6 was directly targeted by miR-144-3p using luciferase reporter assay. Immunohistochemical staining and qRT-PCR assays indicated that BCL6 was up-regulated in human CRC tissues compared with its adjacent non-tumor tissues. Additionally, there was a negative correlation between miR-144-3p and BCL6 in 20 pairs of CRC tissues. Furthermore, we found that BCL6 was a mediator of miR-144-3p repression of cell proliferation and cell cycle arrest in CRC cells.

Wnt/β-catenin signaling is crucial for regulating growth-related pathologies in cancer, being closely related to the development of cancer [29, 30]. Recently, many reports have shown that the Wnt/β-catenin pathway is involved in the progression of CRC through multiple ways [31–33]. In our study, we found that miR-144-3p down-regulated the expression of β-catenin, c-Myc and cyclin D1, which are downstream targets of Wnt signaling in HCT116 cells. Moreover, the ectopic expression of BCL6 restored the inhibition of the expression of β-catenin, c-Myc and cyclin D1 in HCT116 cells, miR-144-3p/BCL6 repressed cell proliferation possibly through Wnt/β-catenin signaling, but the specific regulatory mechanism of miR-144-3p/BCL6 in Wnt/β-catenin signaling requires further study.

In all, our data reveal that miR-144-3p inhibits malignant cell phenotype and Wnt/β-catenin signaling in CRC cells. We demonstrated that BCL6 is targeted and downregulated by miR-144-3p. miR-144-3p repression of malignant cell phenotype and Wnt/β-catenin signaling is mediated by BCL6 in CRC cells. These findings may draw attention to understanding the molecular mechanism of CRC progression and may improve the preventive and therapeutic strategies for CRC.

## Conclusion

Our results show that miR-144-3p is downregulated and related to the clinicopathologic characteristics of patients in CRC tissues. Moreover, miR-144-3p inhibits cell proliferation of colorectal cancer cells by targeting BCL6, possibly through inhibition of Wnt/β-catenin signaling. These findings provide further evidence for the miR-144-3p/BCL6 axis as a therapeutic molecular target for CRC.

## Abbreviations

3′UTR: Three prime untranslated region
BCL6: B-cell lymphoma 6
cDNA: complementary DNA
CRC: Colorectal cancer
miR-144-3p: microRNA-144-3p
miRNA: microRNA
MTT: 3-(4,5-cimethylthiazol-2-yl)-2,5-diphenyl tetrazolium bromide

## Availability of data and materials

The sequence of precursors and mature hsa-miR-144-3p is available in miRBase (http://www.mirbase.org/) under the accession number MIMAT0000436. Any additional information related to this study is available from the author for correspondence upon reasonable request.
